# Genetic variation in the microsomal triglyceride transfer protein (−493G/T) is associated with hepatic steatosis in patients infected with hepatitis C virus

**DOI:** 10.1186/s12879-017-2340-x

**Published:** 2017-03-29

**Authors:** Mariana Cavalheiro Magri, Thamiris Vaz Gago Prata, Caroline Manchiero, Bianca Peixoto Dantas, Celso Carmo Mazza, Fátima Mitiko Tengan

**Affiliations:** 1 Laboratório de Investigação Médica em Hepatologia por Vírus (LIM-47), Faculdade de Medicina, Universidade de São Paulo, Av. Dr. Enéas de Carvalho Aguiar, 470 - Instituto de Medicina Tropical - Prédio 2, 1º andar - sala 106. Bairro Cerqueira César, Sao Paulo, SP CEP 05403-000 Brazil; 2 Departamento de Moléstias Infecciosas e Parasitárias, Hospital das Clínicas. Faculdade de Medicina, Universidade de São Paulo, Sao Paulo, SP Brazil

**Keywords:** Hepatic steatosis, Chronic hepatitis C, Microsomal triglyceride transfer protein (MTTP), Single nucleotide polymorphism (SNP)

## Abstract

**Background:**

In chronic hepatitis C, the fibrosis progression rates are extremely variable and can be influenced by factors associated with the host, virus and environment. Among the associated metabolic factors, hepatic steatosis is characterized by an accumulation of triglycerides in hepatocytes. In the host, genetic determinants of hepatic steatosis are observed, such as single-nucleotide polymorphisms (SNPs) in the microsomal triglyceride transfer protein (MTTP) gene. The MTTP -493G/T SNP appears to play an important role in the regulation of gene expression and influences the plasma concentration of circulating low-density lipoprotein (LDL). The present study investigated the influence of this SNP in the development of hepatic steatosis in patients with chronic hepatitis C and evaluated the association of hepatic steatosis with certain characteristics of these patients and the hepatitis C virus (HCV).

**Methods:**

Two hundred thirty-nine patients with chronic hepatitis C were genotyped for the MTTP -493G⁄T SNP by a polymerase chain reaction-restriction fragment length polymorphism (PCR-RFLP) assay. The association between hepatic steatosis and selected characteristics of the patient and virus was evaluated using bivariate and multivariate analyses.

**Results:**

The most prevalent MTTP -493G/T genotype was GG (46%) followed by GT (43.5%) and TT (10.5%). Multivariate analysis of the total cohort revealed associations between the presence of hepatic steatosis and inflammatory activity of moderate to high intensity (*P* < 0.001), advanced age (*P* = 0.010), elevated gamma glutamyl transpeptidase (GGT) levels (*P* = 0.010) and low LDL levels (*P* = 0.022). Hepatic steatosis was also associated with the TT/GT genotype of the MTTP -493G⁄T SNP in patients infected with HCV genotype 3 (*P* < 0.001).

**Conclusions:**

In chronic hepatitis C patients infected with HCV genotype 3 and with the TT/GT genotype of the MTTP -493G/T SNP, a significant increase in hepatic steatosis was observed, which may indicate that this SNP has a significant influence on the accumulation of triglycerides in hepatocytes. Furthermore, associations were observed between hepatic steatosis and inflammatory activity of moderate to high intensity, advanced age, elevated GGT and low LDL levels.

## Background

The main characteristic of hepatitis C is liver inflammation caused by the hepatitis C virus (HCV). Approximately 3% of the world’s population may be infected with HCV, which indicates that 170 million people are at risk of developing chronic liver disease [1]. HCV genotype 1 is the most prevalent genotype worldwide and accounts for 83.4 million cases, and it is followed by genotype 3 [2]. HCV infection is considered chronic after 6 months of HCV-RNA persistence in the blood. The fibrosis progression rates are extremely variable and can be influenced by factors associated with the host, virus and environment [3].

Hepatic steatosis is a metabolic factor characterized by an accumulation of triglycerides in hepatocytes, and this disorder has a significant impact on the progression of liver fibrosis. The prevalence of steatosis in patients with chronic hepatitis C is approximately 40% [4]. The genotype of the virus may influence the likelihood of developing steatosis, particularly HCV genotype 3, which is associated with a more prevalent and severe steatosis relative to the genotype non-3 [5]. Within HCV-seroconverted subjects, greater changes in the lipid fractions were generally observed in patients with HCV genotype 3 [6]. In the host, genetic determinants of hepatic steatosis are observed such as single-nucleotide polymorphisms (SNPs) in the microsomal triglyceride transfer protein (MTTP) gene and patatin-like phospholipase-3 (PNPLA3) gene [5, 7].

The function of MTTP is related to the assembly and secretion of low-density lipoproteins (LDLs), such as very low-density lipoproteins (VLDLs) [5, 8, 9]. A functional SNP in the promoter region of MTTP (−493G/T) has been found to play an important role in the regulation of gene expression and influences the concentration of circulating LDL in the plasma of healthy individuals [10]. Zheng et al. [11] and Li et al. [12] conducted a meta-analysis and concluded that the MTTP -493G/T SNP is strongly associated with an increased risk of hepatic steatosis, which may contribute to the development of non-alcoholic fatty liver disease (NAFLD). Corroborating these data, Hsiao et al. [13] found that MTTP SNPs, including MTTP -493G/T, could modulate lipid homeostasis and influence serum lipid levels and NAFLD risk. However, the association between the MTTP -493G/T SNP and the pathogenesis of steatosis associated with HCV has not been established. The present study investigated the influence of this polymorphism in the development of hepatic steatosis in patients with chronic hepatitis C from São Paulo, Brazil and evaluated the association between hepatic steatosis and specific characteristics of these patients and HCV.

## Methods

### Study population

A retrospective study with 239 patients chronically infected with HCV was conducted at the Clinical Hospital of the School of Medicine, University of Sao Paulo (HC-FMUSP), Sao Paulo, Brazil from January 2010 to December 2012.

All patients presented with HCV-RNA, had undergone a liver fragment histopathological analysis within the previous three years, were older than 18 years and were not co-infected with hepatitis B virus (HBV) or acquired human immunodeficiency virus (HIV). The study protocol conformed to the ethical guidelines of the 1975 Declaration of Helsinki and was performed according to the recommendations of the ethics committee of HC-FMUSP.

### Clinical and laboratory assessments

The diagnosis of HCV was established based on a qualitative HCV-RNA nested PCR. The serum levels of HCV-RNA were evaluated using the Amplicor HCV Monitor 2.0 test (Roche Diagnostic Systems, Branchburg, New Jersey, USA), and the HCV genotypes were determined by the Line Probe Assay using Versant HCV Genotype 2.0 (Immunogenetics, Ghent, Belgium) according to standard methods.

An interview to evaluate the demographic data and potential risk factors for acquiring the virus as well as a medical consultation and the collection of 10 ml of peripheral blood were conducted from 2010 to 2012. The following characteristics were investigated: age, gender, weight, height, and alcohol and tobacco consumption. The body mass index (BMI) was calculated as the weight divided by the height squared (kg/m^2^).

The biochemical tests included the following: alanine aminotransferase (ALT) level, which was considered elevated at ≥41 U/L; aspartate aminotransferase (AST) level, which was considered elevated at ≥37 U/L; gamma glutamyl transpeptidase (GGT) level, which was considered elevated at ≥61 U/L; triglyceride level, which was considered elevated at >200 mg/dL; total cholesterol level, which was considered elevated at >200 mg/dL; LDL level, which was considered elevated at >130 mg/dL; high-density lipoprotein (HDL) level, which was considered low at <60 mg/dL; and VLDL level, which was considered elevated at >40 mg/dL.

The fibrosis stage and inflammatory activity of the liver were evaluated according to the METAVIR score [14]. The presence of steatosis was evaluated as described by Kleiner et al. [15]. Perls’ staining with score of 0–4 was applied to assess hepatic siderosis. Based on data from the insulin secretion and fasting glucose analyses, the HOMA-IR (homeostasis model assessment of insulin resistance) index of the patients was calculated and patients who presented HOMA-IR ≥3.0 were considered to have insulin resistance [16, 17].

### SNP genotyping

Genomic DNA was extracted from 200 μL of serum using a PureLink Genomic DNA Mini kit (Invitrogen - Life Technologies, Carlsbad, California, USA) according to the manufacturer’s instructions. The SNP rs1800591 is located at the position -493G/T in the promoter region of the MTTP gene on human chromosome 4. For genotyping, we performed polymerase chain reaction-restriction fragment length polymorphism (PCR-RFLP). The protocols were based on the descriptions of Karpe et al. [10] and Bernard et al. [18], although certain thermal cycling conditions and reagents were adjusted. The following primers were employed in the PCR assay: forward 5′ AGTTTCACACATAAGGACAATCATCTA 3′ and reverse 5′ GGATTTAAATTTAAACTGTTAATTCATATCAC 3′. Amplification by PCR was conducted in a final volume of 16.7 μL of reaction mix containing 8.3 μL of GoTaq Green (Promega, Fitchburg, Wisconsin, USA), 5.3 μL of ultrapure water, 0.7 μL of each primer and 1.7 μL of DNA input. The annealing temperature was adjusted to 55 °C. The PCR assay amplified a fragment of 109 bp, and then 15 μL of this amplicon was incubated with 1 μL of the endonuclease restriction enzyme Hph1 (Hph1, 5000 U/ml; New England BioLabs, Ipswich, Massachusetts, USA), 2 μL of buffer (1× NEBuffer 4) and 2 μL of ultrapure water under the following thermocycler conditions: 37 °C for 3 h and inactivation at 65 °C for 20 min. The digested PCR products were subjected to horizontal 4% agarose gel electrophoresis with 6 μL of Diamond Dye (Promega, Fitchburg, Wisconsin, USA) in 0.5× TBE buffer at 120 V for 3 h and 30 min and were analyzed using a UVIdoc HD2 Imaging System (Uvitec Cambridge, Cambridge, United Kingdom). In the presence of the wild-type allele (G), the restriction enzyme cleaves the 109 bp fragment into two fragments of 89 bp and 20 bp. In the presence of the mutated allele (T), the enzyme cleavage site is eliminated, and the full-length fragment (109 bp) remains intact.

### Statistical analysis

The frequencies of steatosis were classified based on personal, clinical and qualitative laboratory characteristics. Quantitative characteristics were classified based on the presence of steatosis using the following measures: mean, standard deviation, median, minimum and maximum [19]. The odds ratio (OR) of each variable with the occurrence of steatosis was estimated with the respective 95% confidence intervals (CI) and bivariate tests using simple logistic regression [20].

A multiple logistic regression model was used [20] for steatosis and the variables that presented levels of significance less than 0.10 (*P* < 0.10) in the bivariate tests. The interaction between the type of HCV genotype and mutation of the MTTP gene was tested, with only the variables with a significance level of less than 0.05 (*P* < 0.05) maintained in the final models.

Qualitative characteristics were classified based on the type of HCV genotype, and associations were verified with the χ^2^ test [19]. The quantitative variables were classified based on the type of HCV genotype and compared using Student’s t-test [19].

The analyses were conducted using the software IBM-SPSS for Windows version 20.0 (Chicago, Illinois, USA), and data were tabulated using the software Microsoft Excel 2003 (Redmond, Washington, USA). Tests were performed with a significance level of 5%.

## Results

For the 239 patients infected with HCV, 55 years was the mean age (range: 29–83 years), 58.2% were female, and 40.6% reported blood transfusion as a potential risk factor for acquiring HCV. Additionally, 19.3% of the patients reported ingesting more than 20 g/day of alcohol and 29.7% of the patients smoked more than 15 packets/day. Regarding the BMI, the majority (39.9%) of the patients were overweight (BMI = 25–29.9). The HCV genotype distributions were as follows: genotype 1 at 78.7%, 2 at 2.9%, 3 at 17.1% and 5 at 1.3%. The distribution of patients according to the pathological characteristics of the liver fragment was as follows: fibrosis 0 at 16.3%, 1 at 40.6%, 2 at 22.2%, 3 at 11.3% and 4 at 9.6%; inflammatory activity 0 at 1.3%, 1 at 33,1%, 2 at 50.6% and 3 at 15.1%; steatosis 0 at 52.3%, 1 at 34,3%, 2 at 11.3% and 3 at 2.1%; siderosis 0 at 78.2%, 1 at 4.2% and 2 at 17.6%.

**Fig. 1:**
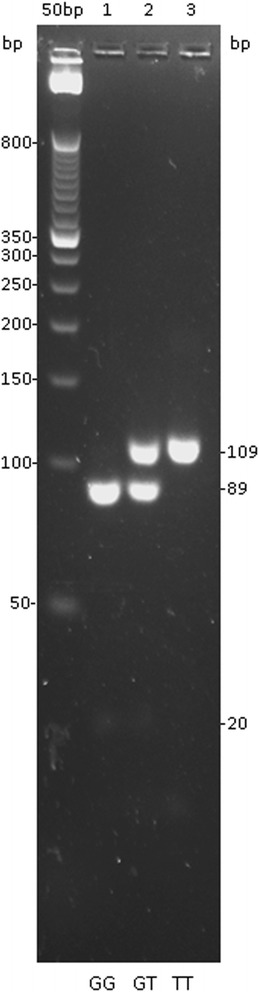
Restriction fragment length polymorphism (RFLP) analysis of the 109 bp amplified product of the MTTP gene. Legend: 4% agarose gel showing: (1) dominant homozygous for the normal GG allele, (2) heterozygous GT, (3) recessive homozygous TT

To genotype the MTTP -493G/T SNP (rs1800591), DNA fragment profiles were generated to identify the normal (wild-type) homozygous genotype (GG), the heterozygous genotype (GT), and homozygous genotype carrying copies of the mutated allele (TT) **(**Figure 1 **)**. The most prevalent genotype was GG (46%) followed by GT (43.5%) and TT (10.5%).

Table 1 shows that the presence of hepatic steatosis alone was significantly more frequent in older patients (*P* = 0.001), in patients with HCV genotype 3 (*P* = 0.029), in patients with advanced fibrosis (grade 3–4) (*P* = 0.002), and in patients with inflammatory activity of moderate to high intensity (grade 2–3) (*P* < 0.001). ALT, AST, GGT and BMI were significantly higher in patients with steatosis (*P* < 0.05), whereas cholesterol and LDL were significantly lower in patients with steatosis (*P* = 0.022 and *P* = 0.005, respectively). HOMA-IR ≥3.0 was associated with the presence of steatosis (*P* = 0.011).

**Table 1:** Description of the occurrence of hepatic steatosis among patients with chronic hepatitis C according to the characteristics evaluated and the results of the bivariate tests

Parameter	Steatosis		OR	95% CI		*P*
	Absent (*n* = 125)	Present (*n* = 114)		Low	Upper	
Age (years)			1.038	1.014	1.061	*0.001*
Mean ± SD	53.1 ± 11.6	58.2 ± 11.8				
Median (min-max)	54 (30–76)	61 (29–83)				
Gender, *n* (%)						0.079
Female	66 (47.5)	73 (52.5)	1.00			
Male	59 (59)	41 (41)	0.63	0.37	1.06	
HCV genotype, *n* (%)						*0.029*
Non-3	110 (55.6)	88 (44.4)	1.00			
3	15 (36.6)	26 (63.4)	2.17	1.08	4.34	
Fibrosis^a^, *n* (%)						*0.002*
0–2	109 (57.7)	80 (42.3)	1.00			
3–4	16 (32)	34 (68)	2.90	1.50	5.61	
Siderosis, *n* (%)						0.491
Absent	100 (53.5)	87 (46.5)	1.00			
Present	25 (48.1)	27 (51.9)	1.24	0.67	2.30	
Inflammatory activity^a^, *n* (%)						*<0.001*
0–1	62 (75.6)	20 (24.4)	1.00			
2–3	63 (40.1)	94 (59.9)	4.63	2.55	8.40	
ALT (U/L)			1.008	1.002	1.014	*0.012*
Mean ± SD	54.1 ± 40.5	70 ± 51.4				
Median (min-max)	44 (11–296)	54.5 (12–311)				
AST (U/L)			1.013	1.005	1.022	*0.003*
Mean ± SD	42.1 ± 30.4	58.8 ± 45.5				
Median (min-max)	33 (14–225)	46 (13–251)				
GGT (U/L)			1.006	1.002	1.009	*0.001*
Mean ± SD	65.6 ± 61.1	113.8 ± 128.8				
Median (min-max)	46 (8–361)	65.5 (10–733)				
Cholesterol (mg/dL)			0.992	0.986	0.999	*0.022*
Mean ± SD	178.7 ± 43.8	166.2 ± 37.1				
Median (min-max)	174 (104–399)	167.5 (69–252)				
LDL (mg/dL)			0.989	0.981	0.997	*0.005*
Mean ± SD	101.8 ± 38.7	88.6 ± 31.1				
Median (min-max)	99 (29–276)	86 (12–176)				
HDL (mg/dL)			1.000	0.986	1.015	0.982
Mean ± SD	55.2 ± 17	55.2 ± 18.1				
Median (min-max)	54 (25–101)	55 (21–110)				
VLDL (mg/dL)			1.004	0.985	1.025	0.661
Mean ± SD	21.7 ± 11.7	22.4 ± 14				
Median (min-max)	19 (7–76)	19 (5–105)				
Triglyceride (mg/dL)			1.001	0.997	1.005	0.670
Mean ± SD	105.9 ± 52.2	109.3 ± 70				
Median (min-max)	97 (34–372)	92.5 (35–502)				
BMI			1.077	1.011	1.147	*0.021*
Mean ± SD	26 ± 3.9	27.3 ± 4.5				
Median (min-max)	25.7 (17.4–38.3)	26.4 (18.5–40.2)				
HOMA-IR, *n* (%)						*0.011*
< 3	88 (59.1)	61 (40.9)	1.00			
≥ 3	34 (41.5)	48 (58.5)	2.04	1.18	3.52	
MTTP, *n* (%)						0.521
GG	60 (54.5)	50 (45.5)	1.00			
GT/TT	65 (50.4)	64 (49.6)	1.18	0.71	1.97	

Bivariate logistic regression,﻿ significance level of *P* < 0.10﻿

^a^Metavir score

Advanced patient age was associated with an increased risk of steatosis; in other words, with each additional year of age the risk increased 4%. Patients with inflammatory activity of moderate to high intensity had a 3.97-fold greater risk of steatosis than patients without inflammatory activity. Elevated GGT levels increased the risk of steatosis, whereas elevated in LDL levels decreased the risk of steatosis. The interaction between the type of HCV genotype and the presence of the MTTP mutation was statistically significant. In patients with HCV genotype 3 and mutation (GT/TT) of the MTTP gene, the risk of steatosis was 25.22-fold greater than in patients without these associated characteristics (*P* < 0.001). Of note, the isolated presence of HCV genotype 3 and the isolated presence of the MTTP mutation did not significantly increase the risk of steatosis (*P* = 0.080 and *P* = 0.116, respectively) (Table 2).

**Table 2:** Multivariate analysis of the characteristics that influenced the presence of hepatic steatosis among patients with chronic hepatitis C

Parameter	OR	95% CI		*P*
		Low	Upper	
Age (years)	1.04	1.01	1.06	**0.010**
HCV Genotype 3	0.30	0.08	1.16	0.080
Inflammatory activity (2–3)	3.97	2.03	7.74	**<0.001**
GGT (U/L)	1.005	1.001	1.009	**0.010**
LDL (mg/dL)	0.990	0.981	0.999	**0.022**
MTTP (GT/TT)	0.60	0.31	1.14	0.116
HCV genotype 3 x MTTP (GT/TT)	25.22	4.24	150.09	**<0.001**

Multiple logistic regression, significance level of *P* < 0.05

### Group analysis: HCV genotype 3 and genotype non-3

The patients were divided into two groups: HCV genotype 3 and genotype non-3 (including genotypes 1, 2 and 5). The study group characteristics are described in Table 3. The analysis of the two groups is presented in Tables 1 and 2.

**Table 3:** Description of characteristics evaluated according to the type of HCV genotype (non-3 and 3) and the results of statistical tests

Parameter	HCV genotype			*P*
	Non-3 (*n* = 198)	3 (*n* = 41)	Total	
Age (years)				0.231
Mean ± SD	55.2 ± 12.3	57.3 ± 9.7	55.5 ± 11.9	
Median (min-max)	57 (29–83)	60 (31–71)	57 (29–83)	
Gender, *n* (%)				0.769
Female	116 (58.6)	23 (56.1)	139 (58.2)	
Male	82 (41.4)	18 (43.9)	100 (41.8)	
Fibrosis^a^, *n* (%)				0.307
0–2	159 (80.3)	30 (73.2)	189 (79.1)	
3–4	39 (19.7)	11 (26.8)	50 (20.9)	
Siderosis, *n* (%)				*0.035*
Absent	160 (80.8)	27 (65.9)	187 (78.2)	
Present	38 (19.2)	14 (34.1)	52 (21.8)	
Inflammatory activity^a^, *n* (%)				0.700
0–1	69 (34.8)	13 (31.7)	82 (34.3)	
2–3	129 (65.2)	28 (68.3)	157 (65.7)	
ALT (U/L)				0.242
Mean ± SD	60.1 ± 44.4	69.5 ± 55.8	61.7 ± 46.6	
Median (min-max)	47 (11–311)	50 (19–296)	47 (11–311)	
AST (U/L)				0.487
Mean ± SD	49.3 ± 40	54 ± 34.9	50.1 ± 39.2	
Median (min-max)	37 (13–251)	46 (21–163)	38 (13–251)	
GGT (U/L)				0.670
Mean ± SD	89.9 ± 99.6	82.4 ± 113.8	88.6 ± 102	
Median (min-max)	56 (8–733)	41 (13–628)	50 (8–733)	
Cholesterol (mg/dL)				*0.007*
Mean ± SD	176 ± 41.4	157 ± 36.4	172.7 ± 41.2	
Median (min-max)	174.5 (69–399)	160 (81–244)	170 (69–399)	
LDL (mg/dL)				*0.024*
Mean ± SD	97.9 ± 36.8	84 ± 28.5	95.5 ± 35.8	
Median (min-max)	97.5 (12–276)	81 (34–157)	93 (12–276)	
HDL (mg/dL)				0.232
Mean ± SD	55.8 ± 18	52.2 ± 14.6	55.2 ± 17.5	
Median (min-max)	55.5 (21–110)	50 (26–89)	54 (21–110)	
VLDL (mg/dL)				0.478
Mean ± SD	22.3 ± 13.2	20.8 ± 10.8	22.1 ± 12.8	
Median (min-max)	19 (5–105)	17 (7–50)	19 (5–105)	
Triglyceride (mg/dL)				0.261
Mean ± SD	109.5 ± 63.4	97.7 ± 48.9	107.5 ± 61.2	
Median (min-max)	95 (34–502)	84 (36–250)	94 (34–502)	
BMI				0.891
Mean ± SD	26.6 ± 4.3	26.5 ± 4.2	26.6 ± 4.2	
Median (min-max)	26.0 (17.4–39.2)	25.7 (18.5–40.2)	25.9 (17.4–40.2)	
HOMA-IR, *n* (%)				0.771
< 3	124 (64.9)	25 (62.5)	149 (64.5)	
≥ 3	67 (35.1)	15 (37.5)	82 (35.5)	
MTTP, *n* (%)				0.183
GG	95 (48)	15 (36.6)	110 (46)	
GT/TT	103 (52)	26 (63.4)	129 (54)	

Associations with qualitative variables were verified with the χ^2^ test, and comparisons of quantitative variables were performed with Student’s t-test, significance level of *P* < 0.10﻿

^a^Metavir score

Patients with HCV genotype 3 had significantly more siderosis than patients with HCV genotype non-3 (*P* = 0.035), and the average cholesterol and LDL levels of patients with HCV genotype 3 were significantly lower (*P* = 0.007 and *P* = 0.024, respectively) (Table 3).

Finally, the multivariable analysis (Table 2) determined the relationship between hepatic steatosis and different MTTP -493G/T SNP genotypes in the patients with HCV genotype 3. Because of the small number of patients with the TT genotype, the patients with the GT and TT genotypes were grouped together into GT/TT group. Forty-one patients were analyzed to investigate the factors associated with steatosis in the patients with HCV genotype 3. An association between genotype GT/TT of the SNP and steatosis was observed in this group of patients.

## Discussion

The results of the present study were used to investigate the relationships among steatosis, chronic hepatitis C, host factors and viral factors. The multivariate analysis of a cohort of 239 Brazilian patients chronically infected with HCV showed that the GT/TT genotype of the MTTP -493G/T SNP (rs1800591) was associated with hepatic steatosis in patients infected with HCV genotype 3, in a total cohort of 239 Brazilian patients chronically infected with HCV, including 198 patients infected with HCV genotype non-3 and 41 infected with HCV genotype 3.

In the bivariate analysis of the total cohort, the presence of hepatic steatosis was associated with HCV genotype 3. The multivariate analysis indicated associations between hepatic steatosis and inflammatory activity of moderate to high intensity, advanced age, elevated GGT and low LDL levels. However, our study presented certain limitations, such as the small number of patients infected with HCV genotype 3, which reduced the statistical power for testing the hypotheses.

The bivariate logistic regression findings regarding the association between hepatic steatosis and HCV genotype 3, age, fibrosis and BMI corroborate the findings of Rubbia-Brandt et al. [21], who evaluated 755 patients with chronic hepatitis C. Among these 755 patients, 178 had HCV genotype 3, and steatosis was independently associated with age (*P* = 0.002), BMI, fibrosis, HCV genotype 3 and alcohol abuse (*P* < 0.001). The authors concluded that hepatic steatosis is associated with liver fibrosis only in patients infected with chronic hepatitis C with HCV genotype 3 and that several factors may determine the progression of liver fibrosis in patients infected with other genotypes, such as age, prolonged alcohol use and possible history of diabetes. In a study with 574 patients with HCV, Patton et al. [22] observed that the degree of steatosis was associated with the BMI, HCV genotype 3, age and duration of infection (*P* < 0.001). In another study of 86 patients who had steatosis, a higher prevalence was observed in HCV genotype 3a infection (*P* < 0.01), and higher serum levels of GGT were observed (*P* < 0.001), consistent with the results of the multivariate analyses in the present study [23]. In addition, the study by Kumar et al. [24] indicated that the HCV genotype 3 (but not genotype 1) plays a direct role in the pathogenesis of hepatic steatosis.

Our results for the associations between hepatic steatosis and HCV genotype 3, age, fibrosis and BMI are also in accordance with those of Leandro et al. [25]. In addition, we identified an association between hepatic steatosis and inflammatory activity in patients infected with HCV (via multiple logistic regression), which is also consistent with the results of Leandro et al. [25], who conducted a robust analysis of 3068 patients contained in 10 databases and concluded that steatosis was associated with fibrosis in patients with chronic hepatitis C via inflammatory reactions because hepatic inflammation can promote fibrogenesis in patients with hepatic steatosis. Inflammatory activity was also associated with hepatic steatosis only in HCV genotype 1, whereas we did not observe this association in the patients included in the HCV genotype non-3 group. In our study, inflammatory activity of moderate to high intensity was present in similar proportions, 65.2 and 68.3%, of the HCV genotype non-3 and genotype 3 groups, respectively. Kobayashi et al. [26] evaluated the clinical and histological statuses and virological characteristics of 256 patients chronically infected with HCV genotypes 1 and 2 to identify the factors associated with ALT serum levels. ALT activity in the liver has been widely used as a marker of liver inflammation. Elevated ALT levels in the serum of the patients evaluated by Kobayashi et al. [26] were strongly associated with severe hepatic inflammation and male gender, and they were also associated with higher HOMA-IR index values, low HDL levels, and a higher degree of hepatic steatosis. The authors hypothesized that specific metabolic factors may originate from the host rather than from the virus.

We observed an association between hepatic steatosis and insulin resistance only in the total cohort of patients infected with HCV by bivariate logistic regression. Fartoux et al. [27] found that insulin resistance was associated with steatosis in patients with HCV genotype 1 but not in patients infected with genotype 3 (*P* = 0.001), which was surprising because steatosis is usually severe in these patients. Fartoux et al. [27] concluded that increasing circulating insulin is a risk factor for fibrosis development in chronic hepatitis C patients because of insulin resistance-induced steatosis. In the present study, insulin resistance was not associated with the viral genotype. A study conducted in Spain with 159 patients showed that insulin resistance was higher in patients infected with genotype 1 than in those infected with genotype 3a (*P* = 0.003), whereas in patients infected with genotype 1, a correlation was observed between insulin resistance and steatosis (*P* = 0.001) [28]. Hui et al. [29] studied 260 patients with chronic hepatitis C, and the independent predictors for the degree of hepatic steatosis were HCV genotype 3 (*P* = 0.001), BMI (*P* = 0.002), portal inflammation (*P* = 0.03) and cholesterol level (a negative association; *P* = 0.004). We also found in bivariate logistic regression that the presence of hepatic steatosis alone was associated with HCV genotype 3, BMI, inflammatory activity of moderate to high intensity, and lower levels of cholesterol and LDL. The inflammatory activity and low LDL levels remained associated with steatosis in multiple logistic regression. Moreover, Hui et al. [29] also found that insulin resistance is an independent predictor for the degree of fibrosis (*P* < 0.001) and fibrosis progression rate (*P* = 0.03). The data found in our study and in the cited studies may support the hypothesis that steatosis is associated with insulin resistance and can contribute to fibrosis progression.

Siderosis did not influence hepatic steatosis, but in the group analysis of HCV genotype 3 and genotype non-3, the presence of siderosis was significantly associated with the HCV genotype 3 group. Adinolfi et al. [23] also observed no association of hepatic iron storage with steatosis, but in contrast to our findings, there was no difference in iron storage between patients infected with HCV genotype 3a and 1a.

Hepatic steatosis was also associated with the GT/TT genotype of the MTTP -493G/T SNP in patients with chronic hepatitis C and HCV genotype 3. In patients with HCV genotype 3 and the MTTP mutation (GT/TT), the risk of steatosis was 25.22 times higher than in patients without these associated characteristics. Zampino et al. [8] and Mirandola et al. [30] evaluated liver biopsies, laboratory tests and MTTP -493G/T SNP presence in patients with chronic hepatitis C and indicated that the MTTP protein has a pathogenic role in the development of fat accumulation in the liver. Zampino et al. [8] found an association between the T allele of the polymorphism and high degrees of fatty liver accumulation in patients infected with HCV genotype 3; conversely, Mirandola et al. [30] found that the GT/TT MTTP genotype was the main risk factor associated with a higher degree of steatosis in HCV genotype non-3. Two other studies reported that the functional -493G/T SNP did not influence the development of hepatic steatosis in patients with chronic hepatitis C and HCV genotype 3 [7, 31]. However, in a study with 174 Brazilian patients from the northeastern region, the G allele was more often present in patients infected with genotype 1 who had higher levels of fibrosis. Additionally, the GG and GT genotypes were considered independent protective factors against steatosis in patients with chronic hepatitis C and HCV genotype 1 and non-1 [32]. Finally, Saad et al. [9] also studied this polymorphism, although not in relation to steatosis, and they reported an association between the GT and TT genotypes and severe fibrosis and cirrhosis (*P* = 0.0001).

## Conclusions

Our results provide evidence that in patients with chronic hepatitis C the interaction between HCV genotype 3 and the GT/TT genotype (as opposed to the GG genotype) of the MTTP -493G/T SNP increases the risk of steatosis by 25.22-fold, which may indicate that this SNP has a significant influence on the accumulation of triglycerides in hepatocytes. Significant associations were also observed between hepatic steatosis and inflammatory activity of moderate to high intensity, advanced age, elevated GGT and low LDL levels. Additional studies on other SNPs of this gene and other genes must be performed to determine whether they are significant risk factors for the predisposition to develop hepatic steatosis in patients with chronic hepatitis C.

## Abbreviations

ALT: Alanine aminotransferase
AST: Aspartate aminotransferase
BMI: Body mass index
CI: Confidence interval
GGT: Gamma glutamyl transpeptidase
HBV: Hepatitis B virus
HCV: Hepatitis C virus
HDL: High-density lipoprotein
HIV: Acquired human immunodeficiency virus
HOMA-IR: Homeostasis model assessment of insulin resistance
LDL: Low-density lipoprotein
MTTP: Microsomal triglyceride transfer protein
NAFLD: Non-alcoholic fatty liver disease
OR: Odds ratio
PCR: Polymerase chain reaction
PNPLA3: Patatin-like phospholipase-3
RFLP: Restriction fragment length polymorphism
SNPs: Single nucleotide polymorphisms
VLDL: Very low-density lipoproteins

## References

[1] Lee MH, Yang H, Yuan Y, L'Italien G, Chen CJ (2014). Epidemiology and natural history of hepatitis C virus infection. World J Gastroenterol.

[2] Messina JP, Humphreys I, Flaxman A, Brown A, Cooke GS, Pybus OG (2015). Global Distribution and Prevalence of Hepatitis C Virus Genotypes. Hepatology.

[3] Westbrook RH, Dusheiko G (2014). Natural history of hepatitis C. J Hepatology..

[4] Negro F, Hepatitis C (2010). Virus-Induced Steatosis: An Overview. Dig Dis.

[5] Adinolfi L, Restivo L, Marrone A (2013). The predictive value of steatosis in hepatitis C virus infection. Expert Rev Gastroenterol Hepatol.

[6] Butt AA, Yan P, Simon TG, Chung RT, Abou-Samra AB; ERCHIVES study team Changes in circulating lipids level over time after acquiring HCV infection: results from ERCHIVES BMC Infect Dis 2015;15:510. doi:10.1186/s12879-015-1268-2. 10.1186/s12879-015-1268-2 PMC4642733 26558512

[7] Cai T, Dufour JF, Muellhaupt B, Gerlach T, Heim M, Moradpour D (2011). Viral genotype-specific role of PNPLA3, PPARG, MTTP, and IL28B in hepatitis C virus-associated steatosis. J Hepatol.

[8] Zampino R, Ingrosso D, Durante-Mangoni E, Capasso R, Tripodi MF, Restivo L (2008). Microsomal triglyceride transfer protein (MTP) –493G/T gene polymorphism contributes to fat liver accumulation in HCV genotype 3 infected patients. J Viral Hepat.

[9] Saad Y, Shaker O, Nassar Y, Ahmad L, Said M, Esmat G (2014). A polymorphism in the microsomal triglyceride transfer protein can predict the response to antiviral therapy in Egyptian patients with chronic hepatitis C virus genotype 4 infection. Gut Liver.

[10] Karpe F, Lundahl B, Ehrenborg E, Eriksson P, Hamsten A (1998). A common functional polymorphism in the promoter region of the microsomal triglyceride transfer protein gene influences plasma LDL levels. Arterioscler Thromb Vasc Biol.

[11] Zheng W, Wang L, Su X, Hu XF (2014). MTP -493 G>T Polymorphism and Susceptibility to Nonalcoholic Fatty Liver Disease: A Meta-Analysis. DNA Cell Biol.

[12] Li L, Wang SJ, Shi K, Chen D, Jia H, Zhu J (2014). Correlation between MTP -493G>T polymorphism and non-alcoholic fatty liver disease risk: a meta-analysis. Genet Mol Res.

[13] Hsiao PJ, Lee MY, Wang YT, Jiang HJ, Lin PC, Yang YH (2015). MTTP-297H polymorphism reduced serum cholesterol but increased risk of non-alcoholic fatty liver disease-a cross-sectional study. BMC Med Genet.

[14] The French METAVIR Cooperative Study Group (1994). Intraobserver and interobserver variations in liver biopsy interpretation in patients with chronic hepatitis C. Hepatology.

[15] Kleiner DE, Brunt EM, Van Natta M, Behling C, Contos MJ, Cummings OW (2005). Design and validation of a histological scoring system for nonalcoholic fatty liver disease. Hepatology.

[16] Moucari R, Asselah T, Cazals-Hatem D, Voitot H, Boyer N, Ripault MP (2008). Insulin resistance in chronic hepatitis C: association with genotypes 1 and 4, serum HCV RNA level, and liver fibrosis. Gastroenterology.

[17] Oliveira LP, Jesus RP, Boulhosa RS, Mendes CM, Lyra AC, Lyra LG (2012). Metabolic syndrome in patients with chronic hepatitis C virus genotype 1 infection who do not have obesity or type 2 diabetes. Clinics (Sao Paulo).

[18] Bernard S, Touzet S, Personne I, Lapras V, Bondon PJ, Berthezene F (2000). Association between microsomal triglyceride transfer protein gene polymorphism and the biological features of liver steatosis in patients with Type II diabetes. Diabetologia.

[19] Kirkwood BR, Sterne JAC (2006). Essential medical statistics.

[20] Hosmer DW, Lemeshow S (2000). Applied Logistic Regression.

[21] Rubbia-Brandt L, Fabris P, Paganin S, Leandro G, Male PJ, Giostra E (2004). Steatosis affects chronic hepatitis C progression in a genotype specific way. Gut.

[22] Patton HM, Patel K, Behling C, Bylund D, Blatt LM, Vallee M (2004). The impact of steatosis on disease progression and early and sustained treatment response in chronic hepatitis C patients. J Hepatology.

[23] Adinolfi L, Gambardella M, Andreana A, Tripodi MF, Utili R, Ruggiero G (2001). Steatosis Accelerates the Progression of Liver Damage of Chronic Hepatitis C Patients and Correlates With Specific HCV Genotype and Visceral Obesity. Hepatology.

[24] Kumar D, Farrell GC, Fung C, George J (2002). Hepatitis C Virus Genotype 3 Is Cytopathic to Hepatocytes: Reversal of Hepatic Steatosis After Sustained Therapeutic Response. Hepatology.

[25] Leandro G, Mangia A, Hui J, Fabris P, Rubbia-Brandt L, Colloredo G (2006). Relationship Between Steatosis, et al. Inflammation, and Fibrosis in Chronic Hepatitis C: A Meta-Analysis of Individual Patient Data. Gastroenterology.

[26] Kobayashi Y, Kawaguchi Y, Mizuta T, Kuwashiro T, Oeda S, Oza N (2011). Metabolic factors are associated with serum alanine aminotransferase levels in patients with chronic hepatitis C. J Gastroenterol.

[27] Fartoux L, Poujol-Robert A (2005). Gue’chot J, Wendum D, Poupon R, Serfaty L. Insulin resistance is a cause of steatosis and fibrosis progression in chronic hepatitis C. Gut.

[28] Romero-Gómez M, Viloria MDM, Andrade RJ, Salmerón J, Diago M, Fernandez-Rodríguez CM (2005). Resistance Impairs Sustained Response Rate to Peginterferon Plus Ribavirin in Chronic Hepatitis C Patients. Gastroenterology.

[29] Hui JM, Sud A, Farrell GC, Bandara P, Byth K, Kench JG (2003). Insulin Resistance Is Associated With Chronic Hepatitis C and Virus Infection Fibrosis Progression. Gastroenterology.

[30] Mirandola S, Osterreicher CH, Marcolongo M, Datz C, Aigner E, Schlabrakowski A (2009). Microsomal triglyceride transfer protein polymorphism (493G/T) is associated with hepatic steatosis in patients with chronic hepatitis C. Liver Int.

[31] Petit JM, Masson D, Minello A, Duvillard L, Galland F, Verges B (2006). Lack of association between microsomal triglyceride transfer protein gene polymorphism and liver steatosis in HCV-infected patients. Mol Genet Metab.

[32] Siqueira ERF, Oliveira CPMS, Correa-Giannella ML, Stefano JT, Cavaleiro AM, Fortes MA (2012). MTP -493G/T gene polymorphism is associated with steatosis in hepatitis C-infected patients. Braz J Med Biol Res.

